# Serological Assessment of Hepatitis in Patients with Inflammatory Bowel Disease in Taiwan: A Retrospective Cohort Analysis

**DOI:** 10.3390/life15060893

**Published:** 2025-05-31

**Authors:** Yueh-An Lee, Hsu-Heng Yen, Yang-Yuan Chen

**Affiliations:** 1 Division of Gastroenterology, Department of Internal Medicine, Changhua Christian Hospital, Changhua 500, Taiwan; 183583@cch.org.tw (Y.-A.L.); 27716@cch.org.tw (Y.-Y.C.); 2 Department of Post-Baccalaureate Medicine, College of Medicine, National Chung Hsing University, Taichung 402, Taiwan

**Keywords:** hepatitis, inflammatory bowel disease, vaccination

## Abstract

Inflammatory bowel disease (IBD), comprising ulcerative colitis (UC) and Crohn’s disease (CD), is a chronic, immune-mediated inflammatory disorder of the gastrointestinal tract. Immunosuppressive therapy administration increases the risk of hepatitis B virus (HBV) and hepatitis C virus (HCV) reactivation. This study aimed to investigate the hepatitis screening rate, serological status, and protective antibody levels among the Taiwanese IBD population. This single-center retrospective study included patients with IBD from January 2016 to December 2024. Hepatitis serological markers were analyzed. Patients were categorized into active HBV infection (HBsAg-positive), resolved HBV infection (HBsAg-negative and anti-HBc-positive), and non-HBV-infected groups, with prevalences of 7.5%, 32.5%, and 0.9%, respectively. This study included 347 patients with IBD (UC: 68.3%; CD: 31.7%), with a mean age of 47.1 ± 16.4 years. Patients born after 1984 demonstrated a significantly reduced HBsAg positivity (0.9% vs. 11.0%; *p* < 0.05) and resolved HBV infection (52.2% vs. 1.0%; *p* < 0.05). However, among non-HBV-infected individuals, only 42.0% had protective anti-HBs levels (≥10 mIU/mL), despite vaccination program initiation. In this study, we found an overall HBsAg positivity rate of 7.5% and an anti-HCV seropositivity rate of 0.9% in our IBD population. Taiwan’s HBV vaccination program has effectively reduced the HBV prevalence. However, a significant proportion of vaccinated individuals lack sufficient protective antibody levels, thereby requiring continued HBV screening and booster vaccinations.

## 1. Introduction

Inflammatory bowel disease (IBD), comprising ulcerative colitis (UC) and Crohn’s disease (CD), is a chronic, immune-mediated inflammatory disorder of the gastrointestinal tract [1,2]. Global IBD incidence and prevalence among adult patients vary markedly by region, with annual incidence ranging from 10.5–46.1/100,000 in Europe, 1.4–1.5/100,000 in Asia and the Middle East, 23.7–39.8/100,000 in Oceania, 0.2–3.7/100,000 in South America, and 7.3–30.2/100,000 in North America, and prevalence ranging from 187–832/100,000 in Europe, 1.8–70.1/100,000 in South America, and 214.9–478.4/100,000 in North America, based on studies conducted between 2000 and 2020 [3]. Over the past decades, the global burden of inflammatory bowel disease (IBD) has markedly increased, with the prevalence exceeding 0.3% in many countries across North America, Europe, and Oceania [4]. While the incidence has stabilized or declined in some Western nations, it continues to rise rapidly in newly industrialized regions such as Asia, South America, and Africa [4,5]. Forecasts also suggest that, driven by natural population growth and increased longevity, the number of individuals living with IBD in Western countries could surpass 10 million by 2030 [5]. The IBD prevalence remains lower in Asia than in Western countries; thus, it is traditionally considered a predominantly Western disease [5,6,7,8,9]. This discrepancy may be attributed to differences in genetic susceptibility and environmental exposures, including westernized diets, sanitation, antibiotic use, and industrialization pace, all of which influence gut microbiota and immune regulation [10,11,12,13]. However, in recent years, an increasing incidence has been observed in Asian regions; for example, a nationwide study in Taiwan from 2001 to 2015 showed that the incidence of Crohn’s disease increased from 0.17 to 0.47 per 100,000, while that of ulcerative colitis rose from 0.54 to 0.95 per 100,000 [5,7,14,15,16,17]. The exact cause of IBD remains unclear; however, it may originate from a complex interaction among genetic predisposition, environmental influences, microbial factors, and immune system dysregulation. Recent studies indicate that dietary Westernization may alter the gut microbiota, potentially contributing to the increasing IBD incidence [18,19].

IBD management involves a combination of pharmacological and nonpharmacological approaches. Medications include 5-aminosalicylic acid (5-ASA), corticosteroids, immunomodulators (such as azathioprine, 6-mercaptopurine, and methotrexate), and biologic agents [20]. Surgical intervention may be required in cases of complications. Furthermore, dietary modifications and lifestyle adjustments play a crucial role in supporting gut health and overall well-being.

However, immunosuppressive therapy administration increases the risk of infections, including hepatitis B virus (HBV) and hepatitis C virus (HCV) reactivation, which causes severe hepatic complications, such as fulminant hepatitis and liver failure [21,22,23]. To mitigate these risks, the current guidelines recommend screening for HBV and HCV before initiating treatment for patients with IBD [20,24,25,26,27,28,29,30].

The 5C Concept (Comprehensive IBD care, Collaboration, Communication, Clinical nurse specialists, and Care pathways) and the 5S Principles (Stage the disease, Stratify patients, Set treatment goals, Select appropriate treatment, and Supervise therapy) for optimal IBD management provide a structured framework to ensure comprehensive care and improved patient outcomes [31].

The prevalence of HBV infection differs significantly globally. Approximately 5% of the population in highly endemic regions, such as Southeast Asia, the Western Pacific, and Africa, has hepatitis B, whereas the prevalence in Europe and the Americas is approximately 1% [32]. The widespread vaccination program has reduced the prevalence of HBV infection [33,34]. However, the status of hepatitis B/C infection in regions with a low IBD incidence but a high hepatitis incidence, such as Taiwan, is less understood [35]. A previous study reported anti-HCV seropositivity in 6.8% of individuals with diabetes mellitus (DM), compared to 2.6% in the general population [36]. However, the prevalence of HCV and HBV infection among patients with inflammatory bowel disease (IBD) remains unclear. This information is clinically important, as patients with IBD receiving immunosuppressive therapy may be at increased risk of hepatitis reactivation. Notably, safe and effective antiviral therapies are currently available for both hepatitis B and C infections.

Therefore, this study aimed to investigate the serological status and protective antibody levels among the central Taiwanese population in a tertiary center.

## 2. Materials and Methods

### 2.1. Study Design

This retrospective observational study included patients diagnosed with IBD, comprising UC and CD, who received follow-up at Changhua Christian Hospital. Data on basic demographic and clinical characteristics were collected, including gender, age, diagnosis, and date of diagnosis. Furthermore, hepatitis serological markers were analyzed, including hepatitis B surface antigen (HBsAg), hepatitis B surface antibody (anti-HBs), hepatitis B core antibody IgG (anti-HBc IgG), and HCV antibody (anti-HCV).

Patients were categorized based on their HBV and HCV serostatus as follows:

- Active HBV infection: positive for HBsAg;
- Resolved HBV infection or occult HBV infection: negative for HBsAg but positive for anti-HBc IgG;
- Non-infected for HBV: negative for both HBsAg and anti-HBc IgG;
- HCV infection: positive for anti-HCV.

This classification enabled a comprehensive assessment of the HBV and HCV prevalence in the IBD population.

### 2.2. Data Source

This study retrospectively analyzed data obtained from patients who attended follow-up visits at the gastroenterology outpatient department of Changhua Christian Hospital, Changhua, Taiwan, a single tertiary center, from January 2016 to December 2024. The hospital was the only medical center in Changhua County, with an area of 1074 km^2^ and a population of 1.2 million, located in central Taiwan. Patients were consecutively recruited, regardless of age, to ensure a comprehensive study population analysis. The Institutional Review Board (IRB no. 250114) of Changhua Christian Hospital approved this study, ensuring compliance with the ethical guidelines and patient confidentiality standards.

### 2.3. Patient Enrollment

Data on age, gender, date of diagnosis, and serological markers for HBV, including HBsAg, anti-HBs Ab, and anti-HBc IgG, as well as HCV markers, specifically anti-HCV, were collected from all patients.

### 2.4. Statistical Analysis

Categorical variables were presented as percentages. The Shapiro–Wilk test was used to assess the normality of continuous variables. As the continuous variables did not follow a normal distribution, they were presented as medians with interquartile ranges (IQRs). The chi-square test was employed for univariate analysis to compare categorical variables. The Statistical Package for the Social Sciences software version 25.0 for Windows and R software version 4.4.1 (2024-06-14) were used for the statistical analysis.

## 3. Results

### 3.1. Baseline Characteristics and Hepatitis Serology Testing Rates in Patients with UC and CD

**Figure 1:**
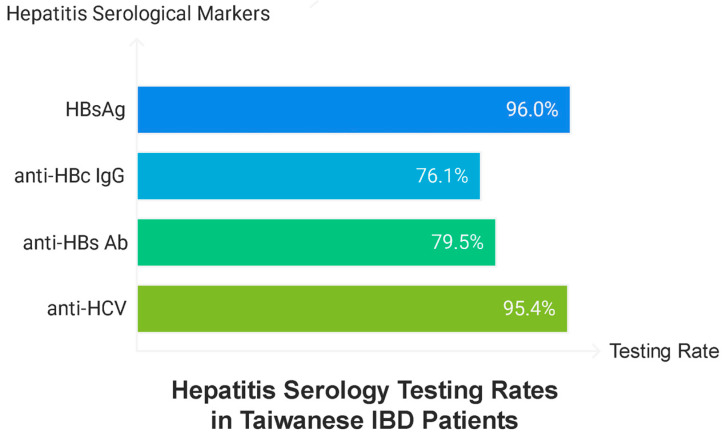
Testing rate of hepatitis serology.

This study included 347 patients diagnosed with IBD, comprising 237 (68.3%) and 110 (31.7%) patients with UC and CD, respectively. The cohort was predominantly male (62.5%), with a median age of 47.2 years (IQR 34.25–58.83). The median age at diagnosis was 37.5 years (IQR 27.67–50.17), with patients with UC being diagnosed at an older age (38.75 years, IQR 30.0–50.33) than those with CD (32.46 years, IQR 23.21–47.73). The testing rate for hepatitis serology was high, with 96.0% of patients undergoing testing for HBsAg, 76.1% for anti-HBc IgG, 79.5% for anti-HBs Ab, and 95.4% for anti-HCV (Figure 1 and Table 1).

### 3.2. Overall Hepatitis Serology Results

**Figure 2:**
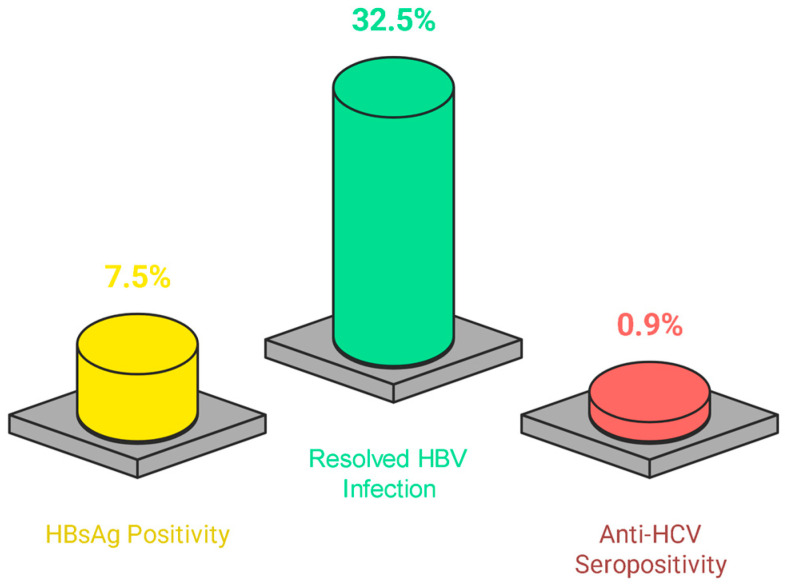
Testing results of hepatitis serology of the study population.

Among all the tested patients, the prevalence of active HBV infection (HBsAg positivity) was 7.5% (25/333). Furthermore, 32.5% (83/255) of the patients experienced resolved HBV infection (anti-HBc IgG positivity and HBsAg negativity), whereas the prevalence of HCV infection (anti-HCV positivity) was 0.9% (3/331) (Figure 2). No cases of HBV–HCV co-infection were observed.

Among patients with resolved HBV infection (n = 83), the majority (83.8%) demonstrated protective anti-HBs levels (≥10 mIU/mL), with 56.8% having high anti-HBs titers (>100 mIU/mL). In contrast, among non-HBV-infected individuals (n = 155), 45.8% exhibited protective anti-HBs levels, and just 18.7% showed titers > 100 mIU/mL.

### 3.3. Impact of Taiwan’s Neonatal HBV Immunization Program

Patients were further categorized into two groups based on birth year: before and after 1984, the year Taiwan implemented its nationwide neonatal HBV immunization program. The rate of HBsAg positivity significantly reduced from 11.0% in those born before 1984 to 0.9% in those born after 1984 (*p* < 0.05). Similarly, the seropositive rate of anti-HBc markedly decreased, from 54.5% in those born before 1984 to 2.0% in those born after 1984 (*p* < 0.05), demonstrating the influence of Taiwan’s nationwide HBV vaccination program. Furthermore, the prevalence of resolved HBV infection significantly decreased from 52.2% before 1984 to 1.0% after 1984 (*p* < 0.05). No significant difference in anti-HCV positivity was observed between the two groups (1.4% vs. 0%; *p* = 0.517). Table 2 shows the results.

Among patients with resolved HBV infection, the proportion with protective anti-HBs antibody levels (≥10 mIU/mL) remained high (84.9% before 1984 vs. 0% after 1984; *p* = 0.356). A lower proportion of individuals with non-HBV infection born after 1984 demonstrated anti-HBs levels of ≥10 mIU/mL compared with those born before 1984 (42.0% vs. 50.7%), but this difference was not statistically significant (*p* = 0.361). A substantial proportion (58%) of individuals from the HBV immunization program (born after 1984) cohort exhibited insufficient protective anti-HBs antibody levels.

## 4. Discussion

Hepatitis B and C can be transmitted from mother to child at birth (perinatal transmission) or through horizontal transmission, including exposure to infected blood, needlestick injuries, tattooing, piercing, and contact with infected bodily fluids. The global prevalence of HBV infection widely differs, with approximately 5% of the population affected in highly endemic regions, such as Southeast Asia, the Western Pacific, and Africa, whereas the prevalence is significantly lower, at approximately 1%, in Europe and the Americas [32]. The prevalence of hepatitis C differs by region, with the highest in the Eastern Mediterranean (1.8%), compared with 0.4% in the Western Pacific and 0.5% in both Southeast Asia and the Americas [32]. Reactivation of this viral hepatitis may cause life-threatening complications during immunosuppressive therapy [34], and knowing a patient’s hepatitis serology status is important for those who may receive immunosuppressive therapy, such as the IBD population. This study revealed that despite the low IBD prevalence in Taiwan, the testing rates for hepatitis were high compared with the previous literature. Furthermore, we demonstrated a decrease in HBV prevalence, particularly in the post-vaccination program population.

Current practice guidelines recommend HBV and HCV screening before initiating treatment for patients with IBD [24,25,26,27,28,29]. Current recommendations stratify patients with chronic and resolved HBV infection into high-risk (reactivation risk ≥ 10%) and moderate-risk (1–10%) categories, respectively, when considering immunosuppressive therapy. Lee et al. [37] from Korea report a large-scale study specifically investigating the clinical course of HBV infection and the risk of liver dysfunction in Asian patients with IBD receiving anti-tumor necrosis factor-α (anti-TNF-α) therapy. In their cohort, the overall incidence of liver dysfunction attributed to HBV reactivation was 7.3%, with a median onset of 32.4 months following the initiation of anti-TNF-α treatment. Importantly, patients with chronic HBV infection exhibited a markedly higher rate of liver dysfunction (15%), whereas liver dysfunction was rare (one case) among those with resolved HBV infection. These results reinforce the notion that anti-TNF-α therapy confers a substantial risk of HBV reactivation-related liver dysfunction in patients with IBD with chronic HBV infection, while the risk in those with resolved infection remains low.

Despite the recommendations of the guidelines, the yield of screening is highly dependent on the adherence of healthcare providers to the practice guidelines, and this step is easily overlooked. Ben et al. conducted a study at a tertiary center in the United States and revealed an HBV screening rate of 51% among patients with IBD from September 2008 to January 2013 [38]. They found that less than half of their patients with IBD had received HBV vaccinations, and the HBV vaccination rates were higher among patients younger than 25 years compared with those over 50 years of age in the United States. This age-dependent difference likely reflects the implementation of routine childhood and adolescent HBV vaccination programs since the early 1990s in the United States. In addition, HBV serologic testing was not routinely performed in a substantial proportion of patients with IBD, and a significant variability was found between different levels of experience among providers, particularly in a teaching hospital setting where trainees are involved in patient care. Similarly, Shah et al., in 2003–2011, recorded an overall HBV screening rate of 23.7% in patients with IBD undergoing anti-TNF treatment [39]. They observed a significant upward trend over time, with screening rates increasing from 8.1% at the beginning of the study to 43.2% by the end. This gradual improvement likely reflects growing awareness and evolving clinical practice patterns, particularly following the release of updated HBV screening guidelines by the Centers for Disease Control and Prevention in 2008, which recommended HBV screening for patients undergoing cytotoxic or immunosuppressive therapy. Several factors were found to influence HBV screening practices. Among patient characteristics, African-American patients were more likely to undergo HBV screening compared with other racial groups, whereas individuals residing in semi-urban or intermediate regions between urban and rural areas were less likely to be screened. Higher screening rates were observed at Veterans Affairs (VA) facilities with academic affiliations and at centers managing a medium-to-high volume of patients with IBD treated with anti-TNF agents. Another study conducted in the Netherlands from 2000 to 2010 demonstrated an increase in screening rates from 36% to 49% in the final two years of the study for patients with IBD receiving anti-TNF therapy [40]. The authors observed a striking discrepancy between the screening rates for tuberculosis and HBV. While TB screening was routinely and consistently performed in the vast majority of patients before the initiation of immunosuppressive therapy, HBV screening was notably underutilized. The high adherence to TB screening protocols may reflect well-established local and international guidelines, as well as the mandatory requirement for TB screening before anti-TNF-α therapy reimbursement. In contrast, HBV screening, although recommended by international guidelines, appears to lack the same level of implementation, especially in Western countries with a low incidence of HBV infection. This discrepancy was attributed to several factors, including lower physician awareness of HBV reactivation risks, the absence of reimbursement-driven mandates for HBV testing, and variability in institutional protocols.

This study revealed a high screening rate for hepatitis status in the whole IBD population, not only in those receiving advanced therapy, compared with previous reports. This may be related to Taiwan’s history as a high-prevalence hepatitis B region and the high awareness of the disease by gastroenterologists in Taiwan. Furthermore, the promotion of the universal hepatitis C elimination program conducted by the government [41] and the dedicated IBD nurse in our hospital for patient care may have contributed to the high screening rate in the present study [42]. This study and previous studies support the Importance of continued efforts to educate healthcare providers about HBV screening, vaccination guidelines, and their relevance in managing patients with IBD, particularly those receiving immunosuppressive therapies.

A recent meta-analysis summarized 34 studies regarding hepatitis serology testing among the IBD population and revealed different patterns in various regions [43]. However, the study did not further analyze the effect of the immunization program. The Taiwanese government implemented a nationwide hepatitis B screening program in July 1984 for pregnant women alongside a neonatal mass immunization program [44]. This initiative successfully reduced the prevalence of chronic HBV carriers and lowered the incidence of hepatocellular carcinoma [45,46]. Furthermore, it significantly curtailed the mother-to-child transmission of HBV, thereby reducing the HBsAg positivity rate from 10.5% to 0.8% [47]. Data for the IBD population are limited, and Chou et al. previously reported that the overall seroprevalence of HBsAg among 158 patients with IBD with available serologic data was 13.3% (CD: 11.3%; UC: 15.4%), closely mirroring the prevalence reported in the general Taiwanese population [35]. The mean age at IBD diagnosis in Chou’s [35] cohort was 38.4 years, indicating that the majority of these patients were born before the introduction of the HBV vaccination program and, therefore, were unable to experience the effect of the vaccination program. This study was the first to investigate the result of the immunization program in the IBD population and observed a comparable trend to the general population, with the HBsAg positivity rate decreasing from 11% before 1984 to 0.9% after 1984, demonstrating the protective effect of the immunization program in the high-endemic area.

Protective antibodies decline over time despite vaccination, with anti-HBs levels decreasing after 10–31 years. The proportion of individuals falling below the protective threshold (10 mIU/mL) differs, with reports ranging from 18% to 62%, according to factors such as age at vaccination and vaccine type [48,49,50]. Studies reveal that an anti-HBs level of >100 mIU/mL may provide stronger and longer-lasting protection against HBV infection [51,52]. This study revealed that 54.2% of the non-HBV-infected population had anti-HBs levels of <10 mIU/mL [53]. The findings of this study are consistent with those from studies conducted in non-IBD Taiwanese populations. Given the waning of anti-HBs titers over time, the administration of a booster dose of the hepatitis B vaccine following primary vaccination should be considered [54].

This study has several limitations. First, this was a retrospective, single-center study, which potentially limits the generalizability of the results to broader IBD populations. Given the lack of existing data on this topic in Taiwan, our findings provide an important foundation for future research evaluating the effectiveness of the hepatitis B vaccination program and the clinical significance of hepatitis B surface antibody titers in patients with IBD. Second, missing laboratory data for some patients, particularly in anti-HBs and anti-HBc IgG testing, could have affected the analysis of HBV serostatus. Furthermore, this study did not assess HBV DNA levels, limiting the ability to detect occult HBV infections and to comprehensively stratify the risk of hepatitis B reactivation. In addition, serial monitoring of hepatitis B antibody levels was not performed across the entire IBD population, which could have provided further insights into immune protection status over time. Despite this limitation, HBV DNA testing and prophylactic antiviral therapy are provided to HBV carriers as part of a risk management program in Taiwan before initiating advanced therapies [34,55]. We did not encounter hepatitis activation-related hospitalization or mortality in this cohort.

## 5. Conclusions

In this study, we found an overall HBsAg positivity rate of 7.5% and an anti-HCV seropositivity rate of 0.9% in our IBD population. Taiwan’s national hepatitis B vaccination program has effectively reduced the prevalence of chronic HBV infection from 11.0% to 0.9%. However, a significant proportion of vaccinated individuals still demonstrated insufficient protective antibody levels, emphasizing the need for continued HBV screening and booster vaccinations for this IBD population.

## Figures and Tables

**Table 1:** Baseline characteristics and hepatitis serology testing rates in patients with UC and CD.

	UC (n = 237)	CD (n = 110)	All Patients (n = 347)	*p*-Value
Sex, male, n (%)	146 (61.6%)	71 (64.5%)	217 (62.5%)	0.6835
Age, year, median (IQR)	49.25 (37.0–58.96)	41.54 (30.52–57.94)	47.17 (34.25–58.83)	0.0083
Age at IBD diagnosis, year, median (IQR)	38.75 (30.0–50.33)	32.46 (23.21–47.73)	37.5 (27.67–50.17)	0.0186
HBsAg test, n (%)	227 (95.8%)	106 (96.4%)	333 (96.0%)	1
Anti-HBc IgG test, n (%)	180 (75.9%)	84 (76.4%)	264 (76.1%)	1
Anti-HBs Ab test, n (%)	199 (84.0%)	77 (70.0%)	276 (79.5%)	0.0043
Anti-HCV test, n (%)	227 (95.8%)	104 (94.5%)	331 (95.4%)	0.8139

**Table 2:** Comparison of hepatitis B and C serological status before and after the implementation of Taiwan’s HBV vaccination policy in 1984.

	Before 1984 (228)	After 1984 (119)	All Patients	*p*-Value
HbsAg (+)	24/219 (11.0%)	1/114 (0.9%)	25/333 (7.5%)	<0.05
Anti-HBc (+)	90/165 (54.5%)	2/99 (2.0%)	92/264 (34.8%)	<0.05
Resolved HBV	82/157 (52.2%)	1/98 (1.0%)	83/255 (32.5%)	<0.05
Anti-HCV (+)	3/218 (1.4%)	0/113 (0%)	3/331 (0.9%)	0.517
Immune Status of Resolved HBV Infection (n = 83) *				
Anti-HBs Antibody Level	Before 1984	After 1984	All patients	*p*-Value
<10 mIU/mL	11/73 (15.1%)	1/1 (100%)	12/74 (16.2%)	0.356
≥10 mIU/mL	62/73 (84.9%)	0%	62/74 (83.8%)	0.356
>100 mIU/mL	42/73 (57.5%)	0%	42/74 (56.8%)	0.891
Immune Status of Non-HBV Infection (n = 172) **				
Anti-HBs Antibody Level	Before 1984	After 1984	All patients	*p*-Value
<10 mIU/mL	33/67 (49.3%)	51/88 (58.0%)	84/155 (54.2%)	0.361
≥10 mIU/mL	34/67 (50.7%)	37/88 (42.0%)	71/155 (45.8%)	0.361
>100 mIU/mL	14/67 (20.9%)	15/88 (17.0%)	29/155 (18.7%)	0.688

* Nine patients born before 1984 did not undergo anti-HBs antibody level testing. ** Eight patients born before 1984 and nine patients born after 1984 did not undergo anti-HBs antibody level testing.

## Data Availability

The data that support the findings of this study are available from the corresponding author upon reasonable request.

## Abbreviations

The following abbreviations are used in this manuscript:

CD	Crohn’s disease
UC	Ulcerative colitis
IBD	Inflammatory bowel disease
HBV	Hepatitis B virus
HCV	Hepatitis C virus
